# Glycoprotein non-metastatic melanoma protein B expression correlates with the prognosis of acute liver injury/failure

**DOI:** 10.3389/fcell.2023.1242152

**Published:** 2023-10-24

**Authors:** Kotaro Kumagai, Shuji Kanmura, Seiichi Mawatari, Yuko Nakamura, Hiromi Eguchi, Oki Taniyama, Ai Toyodome, Sho Ijuin, Haruka Sakae, Kazuaki Tabu, Kohei Oda, Keita Shimata, Taizo Hibi, Akio Ido

**Affiliations:** ^1^ Digestive and Lifestyle Diseases, Department of Human and Environmental Sciences, Kagoshima University Graduate School of Medical and Dental Sciences, Kagoshima, Japan; ^2^ Department of Pediatric Surgery and Transplantation, Kumamoto University Graduate School of Medical Sciences, Kumamoto, Japan

**Keywords:** acute liver failure, prognosis, GPNMB, macrophage, M2c macrophage

## Abstract

**Background:** Glycoprotein non-metastatic melanoma protein B (GPNMB) is expressed in macrophages during recovery from acute liver injury (ALI) in carbon tetrachloride (CCl_4_)-induced liver injury model mice. In this retrospective study, we assessed whether GPNMB levels in the serum and injured liver correlate with liver injury severity and prognosis in patients with ALI or acute liver failure (ALF).

**Methods:** The study involved 56 patients with ALI or ALF who visited the Kagoshima University Hospital. Serum GPNMB level was measured over time, and the localization, proportion, origin, and phenotype of GPNMB-expressing cells in the injured liver were assessed. Finally, the phenotypes of human monocyte-derived macrophages and peripheral blood mononuclear cells (PBMCs) of patients with ALI and ALF were analyzed.

**Results:** Peak GPNMB levels were significantly higher in patients with ALF and hepatic encephalopathy (HE), as well as in those who underwent liver transplantation or died, than in others. The peak GPNMB level correlated with prothrombin activity, prothrombin time-international normalized ratio, Model for End-stage Liver Disease score, and serum hepatocyte growth factor level. GPNMB was expressed in CD68-positive macrophages, and its level increased with the severity of liver injury. The macrophages showed the same polarization as M2c macrophages induced with interleukin-10 from human monocytes. Moreover, PBMCs from patients with ALF exhibited an immunosuppressive phenotype.

**Conclusion:** We found that GPNMB levels in the serum and injured liver, which increased in patients with ALF, especially in those with HE, correlated with the severity of liver injury and prognosis of ALI and ALF. Moreover, GPNMB-positive macrophages exhibited the M2c phenotype. Our results indicate that persistently high GPNMB levels may be a prognostic marker in patients with ALI and ALF.

## 1 Introduction

Acute liver failure (ALF) is a life-threatening critical illness (Bernal and Wendon, 2013). However, to date, there is no effective pharmaceutical treatment to improve the outcome of patients with ALF, except for N-acetylcysteine for acetaminophen hepatotoxicity (Lee et al., 2012). Liver transplantation (LT) remains the only established treatment for patients with ALF. To optimize the effective use of LT, The Model for End-stage Liver Disease (MELD) and MELD-Na scores are used to prioritize patients awaiting LT (Kamath et al., 2001; Kim et al., 2008). Additionally, these scores are correlated with the prognosis of patients with ALF. However, in certain countries, such as Japan, the availability of liver donors is limited (Yoshimura et al., 2010). Therefore, to address the urgent need for novel effective therapies for ALF, the mechanism underlying ALF should be elucidated.

Glycoprotein non-metastatic melanoma protein B (GPNMB) is a transmembrane glycoprotein originally identified in human melanoma cells (Weterman et al., 1995). It is also known as osteoactivin (Safadi et al., 2001) or dendritic cell heparin sulfate proteoglycan integrin-dependent ligand (Shikano et al., 2001). GPNMB is directed to the cell membrane and is released as a soluble fragment cleaved by the metalloproteinase ADAM10 (Rose et al., 2010). It is highly expressed in cancers such as glioma, melanoma, and breast, lung, and stomach cancers (Li et al., 2014; Li et al., 2020; Saade et al., 2021). Moreover, GPNMB overexpression in hepatocellular carcinoma promotes invasion and metastasis (Onaga et al., 2003). GPNMB is expressed by immune cells, including macrophages and dendritic cells (Ahn et al., 2002; Zhuo and Zhou, 2016; Saade et al., 2021); it promotes the maturation of lymphohematopoietic stem cells (Bandari et al., 2003) and decreases the activation of T lymphocytes (Chung et al., 2007). Additionally, GPNMB is expressed by macrophages during recovery from acute liver injury (ALI); these cells have high phagocytic activity and contribute to the balance between fibrosis and fibrolysis in a carbon tetrachloride (CCl_4_)-induced liver injury mouse model (Kumagai et al., 2015). In humans, serum GPNMB level is also increased in non-alcoholic steatohepatitis and alcohol-associated hepatitis (AH) (Katayama et al., 2015; Harris et al., 2022). GPNMB is also expressed by CD68-positive macrophages in cirrhotic human liver (Ramachandran et al., 2012). However, to the best of our knowledge, there are no studies on the serum GPNMB level in patients with ALI and ALF, and its implication for prognosis has not been clarified. Thus, the present study aimed to investigate whether the dynamics of GPNMB levels in the serum and GPNMB expression in injured liver tissues of patients with ALI and ALF reflect prognosis. We also aimed to explore the clinical implications of GPNMB in these patients.

## 2 Methods

### 2.1 Study population

From 1 June 2006, to 30 December 2018, 193 patients with ALI or ALF visited Kagoshima University Hospital, Kagoshima, Japan. Data from 56 of these patients whose serum GPNMB level was measured at least three times during the observation period were retrospectively analyzed. The study protocol conformed to the ethical guidelines of the Declaration of Helsinki and was approved by the Kagoshima University Hospital Clinical Research Ethics Committee (approval numbers: 180320, 180287). Informed consent was obtained from patients or appropriate family members in the form of opt-out on the website.

### 2.2 Definitions and criteria

The day on which patients had peak alanine aminotransferase (ALT) levels during the observational period was considered day 0. The observational period spanned from symptom onset to the day when the patients recovered and were discharged. The outcomes included survival, LT, or death. Patients with ALT levels twice the upper limit of normal and no history of liver disease were defined as having ALI. ALF was defined as a prothrombin time-international normalized ratio (INR) of ≥1.5 in a patient without a history of liver disease, with or without hepatic encephalopathy (HE), in accordance with the diagnostic criteria for ALF and the classification of HE published by the Intractable Hepato-Biliary Diseases Study Group of Japan (Sugawara et al., 2012) and West Haven Criteria (Vilstrup et al., 2014). MELD score was calculated using the following formula, based on the hematological examination results (Kamath et al., 2001): MELD = 9.57 log_e_ (Cre [mg/dL]) + 3.78 log_e_ (total bilirubin [mg/dL]) + 11.20 log_e_ (INR) + 6.43.

### 2.3 Measurement of serum GPNMB and hepatocyte growth factor (HGF) levels

Serum GPNMB and HGF levels were measured using the Human Osteoactivin/GPNMB DuoSet ELISA (DY2550, R&D Systems, Minneapolis, MN, United States) and Enzyme Immunoassay (BML, INC., Tokyo, Japan) according to the manufacturer’s protocol, respectively.

### 2.4 Histological analysis

Hematoxylin-eosin staining (MUTO PURE CHEMICALS CO., LTD., Tokyo, Japan) was performed according to the manufacturer’s protocol. For immunohistochemical analysis, liver specimens were fixed in 10% buffered formalin and incubated with anti-CD68 (Clone: PG-M1; DAKO, Glostrup, Denmark) and anti-GPNMB (Polyclone; R&D Systems) antibodies at 4°C overnight, followed by incubation with peroxidase-labeled anti-mouse or anti-goat antibodies (Histofine Simple Stain MAX PO; Nichirei, Tokyo, Japan) at 20°C for 35 min. For immunofluorescence staining, the paraffin-embedded tissue sections were incubated with primary antibodies at 4°C overnight, followed by incubation with a secondary antibody conjugated with a fluorophore (Supplementary Table S2) at 20°C for 35 min. A BZ-9000 microscope (KEYENCE, Osaka, Japan) was used to capture and analyze fields at ×200 or ×400 magnification. Image analysis was performed using ImageJ ver. 1.51 software (National Institutes of Health, Bethesda, MD, United States).

### 2.5 Isolation of human monocytes from peripheral blood mononuclear cells (PBMCs) and macrophage differentiation

PBMCs were obtained from a healthy volunteer and centrifuged (20°C, 2,200 rpm, 22 min) over a density gradient medium (Histopaque^®^-1,077; Sigma-Aldrich, St. Louis, MO, United States). Human monocytes were isolated from PBMCs using the EasySep™ Human Monocyte Isolation Kit (STEMCELL Technologies, Vancouver, Canada; Catalog #19359) according to the manufacturer’s instructions. The cells were washed, resuspended in ImmunoCult™-SF Macrophage Medium with 25 ng/mL human recombinant macrophage colony-stimulating factor (STEMCELL Technologies; Catalog #78057), seeded in 12-well plates (1 × 10^6^ cells per well), cultured for 6 days, and defined as M0 macrophages. For macrophage activation, the cells were treated for 2 days with 20 ng/mL interferon-gamma (PeproTech, Rocky Hill, NJ, United States) and 10 ng/mL lipopolysaccharide (LPS) (Sigma-Aldrich) to induce M1 macrophages, 20 ng/mL interleukin (IL)-4 (PeproTech) to induce M2a, 10 ng/mL IL-1β (PeproTech) and 10 ng/mL LPS to induce M2b, and 10 ng/mL IL-10 (PeproTech) to induce M2c macrophages in DMEM containing 10% fetal calf serum and 1% penicillin–streptomycin.

### 2.6 RNA isolation and reverse transcription quantitative PCR (RT-qPCR)

Total RNA was extracted from cells using TRIzol reagent (Thermo Fisher Scientific, Waltham, MA, United States). First-strand cDNA was synthesized from 500 ng of total RNA using the PrimeScript RT Master Mix (Takara Bio Inc., Shiga, Japan). RT-qPCR was performed with TB Green Premix Ex Taq II (Takara Bio Inc.) using an ABI Prism 7,700 sequence detection system (Applied Biosystems, Foster City, CA, United States). Data were collected and analyzed using the StepOnePlus Real-Time PCR System (Applied Biosystems). After detecting the threshold cycle for each mRNA in each sample, we calculated the relative concentrations and normalized them to the *β-actin* level. The PCR conditions included an initial denaturation period at 95°C for 30 s, followed by a two-step PCR program at 95°C for 5 s and 60°C for 34 s for 40 cycles. All reactions were performed in duplicates using human primer sequences (Supplementary Table S3).

### 2.7 Flow cytometry

The cells were harvested using a scraper and centrifuged for collection (4°C, 1,600 rpm, 4 min). After Fc receptor blockade (Human TruStain FcX™; BioLegend, San Diego, CA, United States), the cells were stained with fluorophore-conjugated antibodies (Supplementary Table S4) for 1 h on ice, washed, resuspended in 4% paraformaldehyde phosphate buffer solution (FUJIFILM Wako Pure Chemical Corporation, Osaka, Japan), and analyzed using a CytoFLEX flow cytometer (Beckman Coulter, Brea, CA, United States) and FlowJo software ver. 10.5.2 (BD, Franklin Lakes, NJ, United States). The gating strategies are shown in Supplementary Figure S3.

### 2.8 Statistical analysis

All data are expressed as median or mean and individual data points. Data were statistically analyzed using the chi-square test, Mann–Whitney *U* test, Spearman’s rank-correlation coefficient, and one-way analysis of variance with appropriate *post hoc* tests (GraphPad Prism version 8.4.0). Results with *p* < 0.05 were considered statistically significant. Independent *in vitro* experiments were performed at least thrice.

## 3 Results

### 3.1 Patient characteristics and outcomes

All patients with ALI (n = 23) and ALF without HE (n = 23) survived; however, only four of the 10 patients with ALF and HE survived, three underwent LT, and the remaining three died (Table 1 and Supplementary Figure S1).

**TABLE 1:** Patient characteristics.

	ALI	ALF without HE	ALF with HE	*p*-value
Median (Min-Max)				
Age (years)	53 (19–83)	60 (22–72)	49 (19–72)	0.8079
Sex (Male/Female)	11/12	6/17	5/5	0.2388
INR	1.11 (0.86–1.43)	1.67 (1.36–2.53)	2.55 (1.57–5.41)	<0.0001
Aspartate aminotransferase (U/L)	617 (45–8,839)	2,924 (176–15335)	519 (229–9,624)	0.0068
Alanine aminotransferase (U/L)	891 (65–6,313)	3,126 (125–8,878)	718 (248–5,681)	0.0210
Total bilirubin (mg/dL)	6.1 (0.6–26.1)	5.2 (1.7–20.0)	9.3 (4.1–25.2)	0.2550
Creatine (mg/dL)	0.69 (0.46–1.91)	0.64 (0.44–2.27)	1.15 (0.45–3.90)	0.2454
MELD score	15 (6–20)	19 (13–31)	39 (33–40)	<0.0001
MELD-Na score	17 (6–22)	22 (12–32)	39 (33–40)	<0.0001
SOFA score	3 (0–5)	3 (1–8)	8 (4–16)	<0.0001
Etiology (Virus (HAV/HBV/HCV/EBV or CMV)/Autoimmune Hepatitis/Drug-induced liver injury/Indeterminate)	8 (2/4/0/0/2)/8/7/0	11 (6/5/0/0/0)/3/9/0	1 (0/0/1/0/0)/0/4/5	<0.0001
Liver failure (Absent/Present)	23/0	0/23	0/10	<0.0001
Development of hepatic encephalopathy (Absent/Present)	23/0	23/0	0/10	<0.0001
Clinical outcome (Survival/Liver transplantation/Death)	23/0/0	23/0/0	4/3/3	<0.0001

MELD, Model for End-Stage Liver Disease; SOFA, sequential organ failure assessment; HAV, hepatitis A virus; HBV, hepatitis B virus; HCV, hepatitis C virus; EBV, Epstein–Barr virus; CMV, cytomegalovirus; ALI, acute liver injury; ALF, acute liver failure; HE, hepatic encephalopathy; INR, international normalized ratio.

### 3.2 Persistently high serum GPNMB level correlates with severity of liver injury and prognosis of patients with ALI and ALF

**FIGURE 1:**
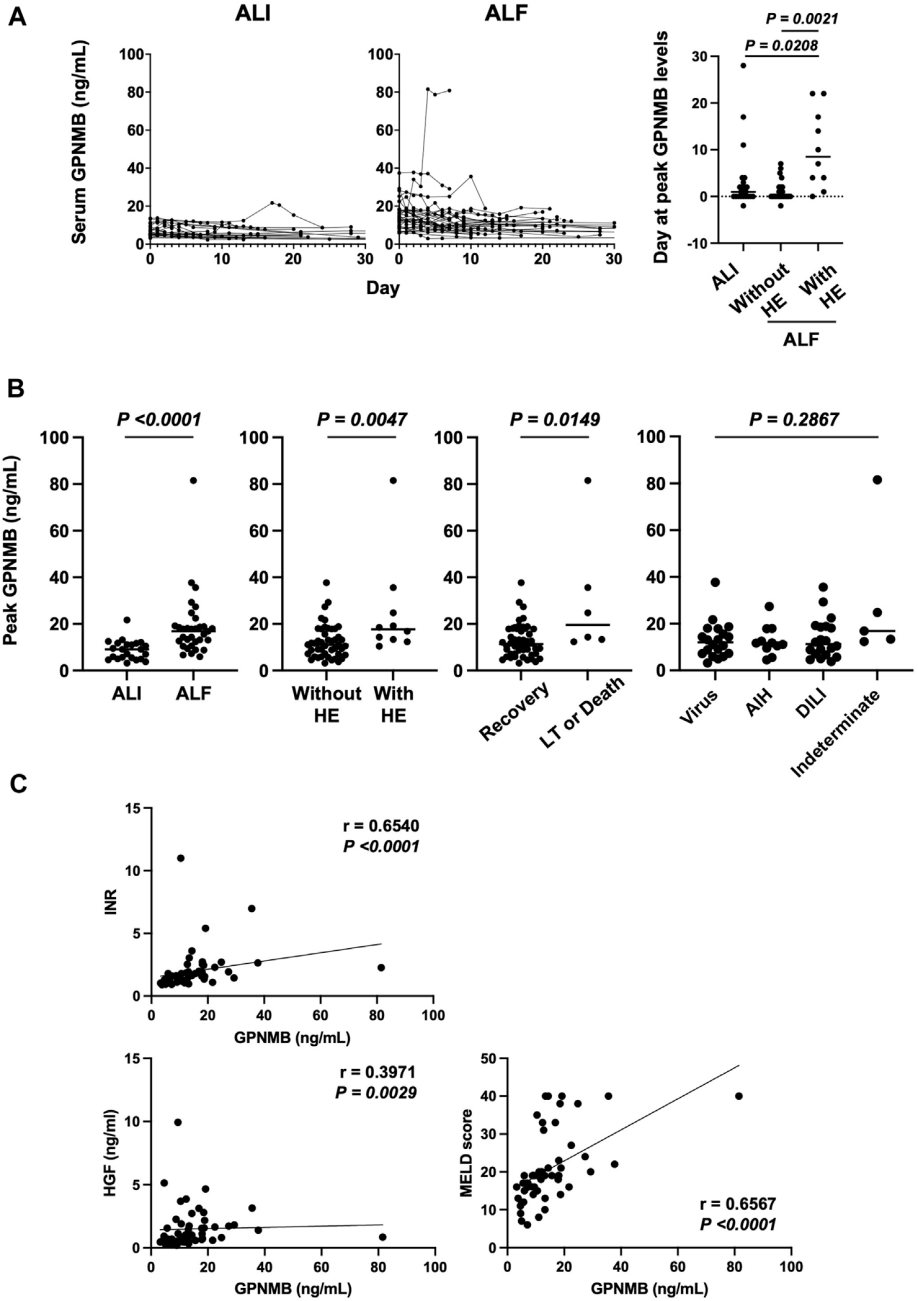
Serum GPNMB level correlates with liver injury severity and prognosis in patients with ALI and ALF. **(A)** Day 0 indicates peak ALT. Time course of serum GPNMB level and day of peak GPNMB level in patients with ALI (n = 23) and ALF (n = 33). **(B)** Comparison of peak serum GPNMB level. ALI (n = 23) vs. ALF (n = 33), patients without HE (n = 46) vs. those with HE (n = 10), recovery (n = 50) vs. LT and death (n = 6), and virus (n = 20) vs. AIH (n = 11) vs. DILI (n = 20) vs. Indeterminate (n = 5). **(C)** Correlation between the peak serum GPNMB level and severity or prognosis of ALI. Mann–Whitney *U* test and Spearman’s rank-correlation coefficient. GPNMB, glycoprotein non-metastatic melanoma protein B; ALI, acute liver injury; ALF, acute liver failure; HE, hepatic encephalopathy; LT, liver transplantation; AIH, autoimmune hepatitis; DILI, drug-induced liver injury.

The serum GPNMB level was higher over time in patients with ALF than in those with ALI. The peak GPNMB level was significantly delayed after a peak in the ALT level in patients with ALF and HE compared with that in patients with ALI (*p* < 0.0208) or ALF without HE (*p* < 0.0021) (Figure 1A). Hence, the peak GPNMB level was significantly higher in patients with ALF (*p* < 0.0001) and HE (*p* = 0.0047), and in those who underwent LT or died (*p* = 0.0149) than in other patients. In contrast, there was no difference in GPNMB levels among different etiologies (Figure 1B). Additionally, the peak GPNMB level significantly correlated with INR (*p* < 0.0001), serum HGF level (*p* = 0.0029), and MELD score (*p* < 0.0001) (Figure 1C). Moreover, both the MELD-Na score and Sequential Organ Failure Assessment (SOFA) score, which is used to assess multiple organ failure, were significantly correlated with the peak GPNMB level (Supplementary Figure S2) (Kim et al., 2008; Singer et al., 2016).

### 3.3 Increased expression of GPNMB and CD68 in injured liver tissues of patients with ALF and HE

**FIGURE 2:**
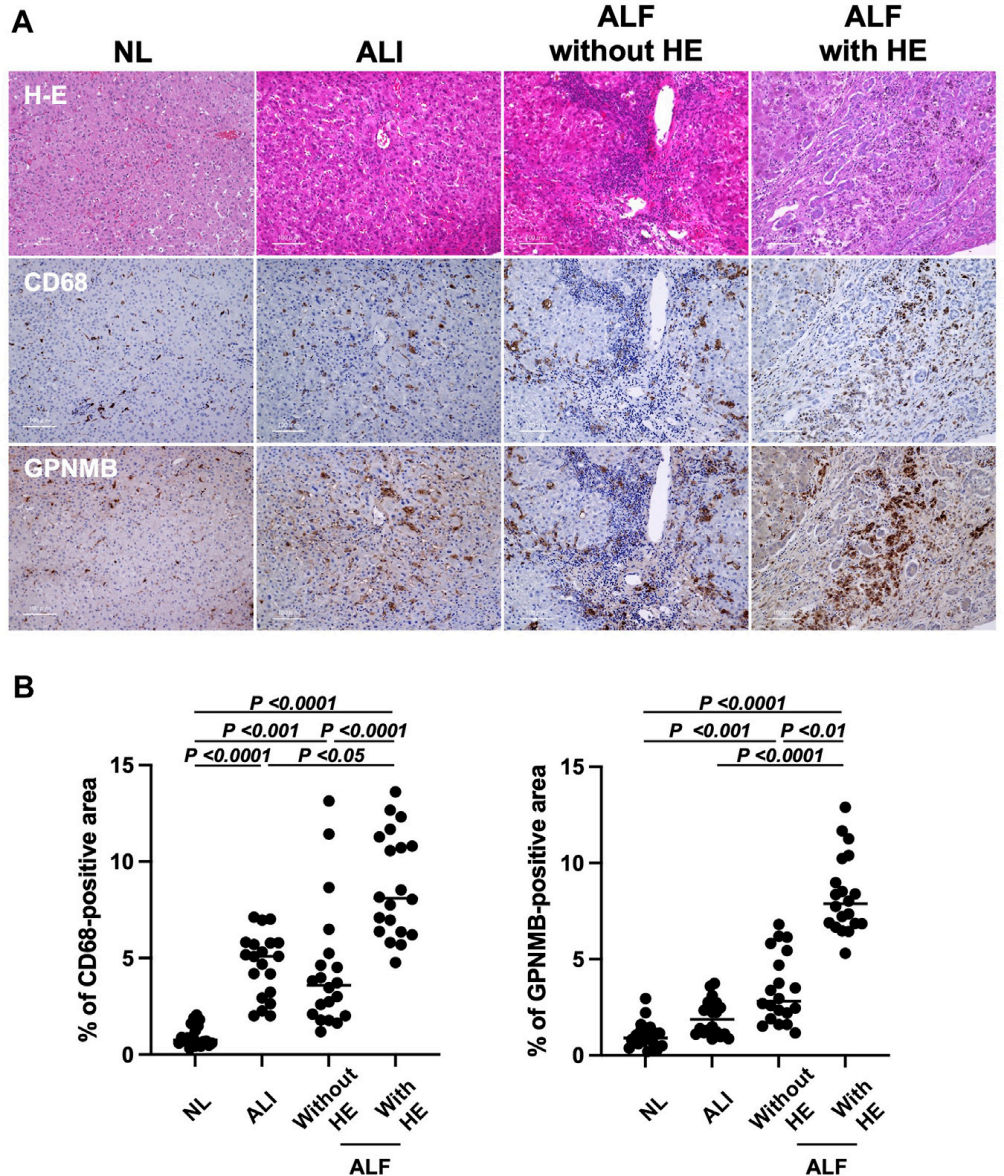
GPNMB and CD68 expression in injured liver tissues correlates with liver injury severity and prognosis in patients with ALI and ALF. **(A)** Liver specimens were fixed in 10% buffered formalin and incubated with anti-CD68 and anti-GPNMB at 4°C overnight, followed by incubation with peroxidase-labeled anti-mouse or anti-goat antibodies at 20°C for 35 min. Representative hematoxylin and eosin, CD68, and GPNMB staining in injured liver tissue sections (×200 magnification; scale bar, 100 µm) for patients with ALI, and ALF with and without HE. As a reference, images of stained normal liver tissues (NL) are displayed. **(B)** Percentage of CD68-positive and GPNMB-positive areas in injured liver tissues of patients with ALI, and ALF with and without HE is represented as individual dot plots, with NL as a reference. For the quantitation of positive area, five fields per individual injured liver tissue (n = 4 per group with 20 dot plots per group) were measured using ImageJ software. One-way analysis of variance was used for statistical comparison. GPNMB, glycoprotein non-metastatic melanoma protein B; ALI, acute liver injury; ALF, acute liver failure; HE, hepatic encephalopathy.

To determine whether serum GPNMB level elevation originated from injured liver tissues in patients with ALI and ALF, we assessed GPNMB expression in the injured liver tissues of these patients. Of the 56 patients, we selected four from each group (ALI, ALF without HE, and ALF with HE) (Supplementary Table S1). GPNMB expression significantly increased with the severity of liver injury (*p* < 0.0001), similar to CD68 expression (*p* < 0.0001), a pan-macrophage marker (Figures 2A, B).

### 3.4 GPNMB is expressed in CD68-Positive M2 macrophages in injured liver tissues of patients with ALF and HE

**FIGURE 3:**
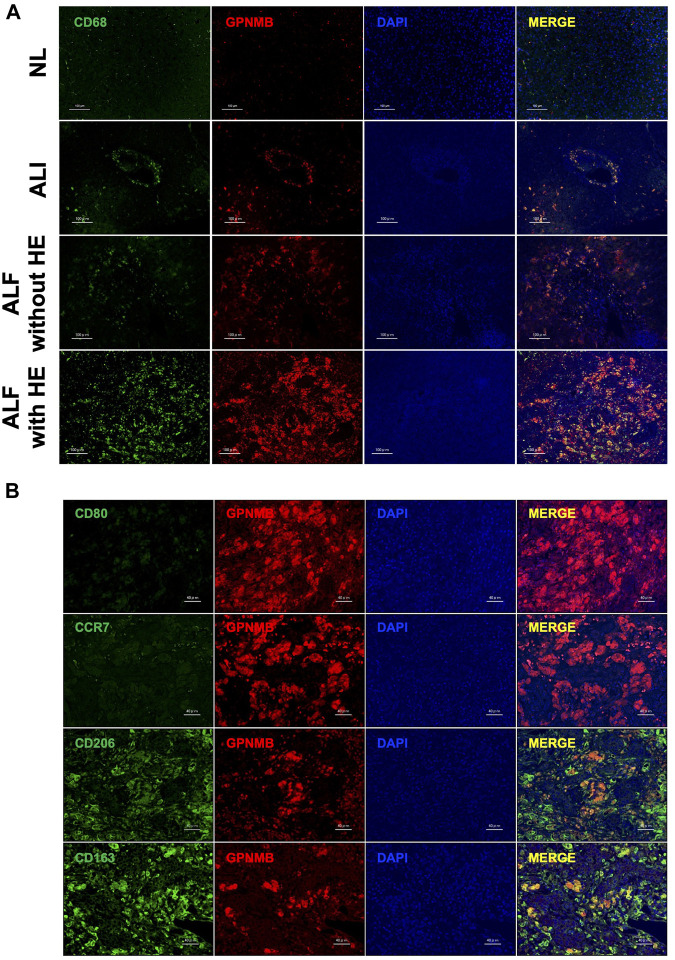
GPNMB is expressed by CD68-positive M2 macrophages in injured liver tissues. **(A)** Localization of GPNMB expression in paraffin-embedded sections of injured liver tissues from patients with ALI, and ALF with and without HE was analyzed using immunofluorescence microscopy at ×200 magnification (scale bars, 100 µm). As a reference, images of stained normal liver tissues (NL) are displayed. CD68, green; GPNMB, red; DAPI, blue. **(B)** Phenotype of GPNMB-positive cells in injured liver tissues of patients with ALF and HE was analyzed using immunofluorescence microscopy at ×400 magnification (scale bars, 40 µm). CD80, CCR7, CD163, and CD206, green; GPNMB, red; DAPI, blue. GPNMB, glycoprotein non-metastatic melanoma protein B; ALI, acute liver injury; ALF, acute liver failure; HE, hepatic encephalopathy; DAPI, 4′,6-diamidino-2-phenylindole; H-E, hematoxylin and eosin.

As GPNMB was expressed in injured liver tissues, we confirmed the phenotype of GPNMB-expressing cells. Regardless of the severity of liver injury, GPNMB was expressed in CD68-positive macrophages (Figure 3A). Additionally, we assessed the phenotype of macrophages in injured liver tissues of patients with ALF and HE, as GPNMB expression was increased in these tissues. GPNMB-positive macrophages did not express CD80 and C-C chemokine receptor (CCR) 7, which are proinflammatory phenotypic markers of M1 macrophages, and expressed CD206 moderately and CD163 strongly, which are anti-inflammatory phenotypic markers of M2 macrophages (Figure 3B).

### 3.5 Human monocyte-derived M2c macrophages induced by IL-10 express GPNMB and CD163 strongly and CD206 moderately

**FIGURE 4:**
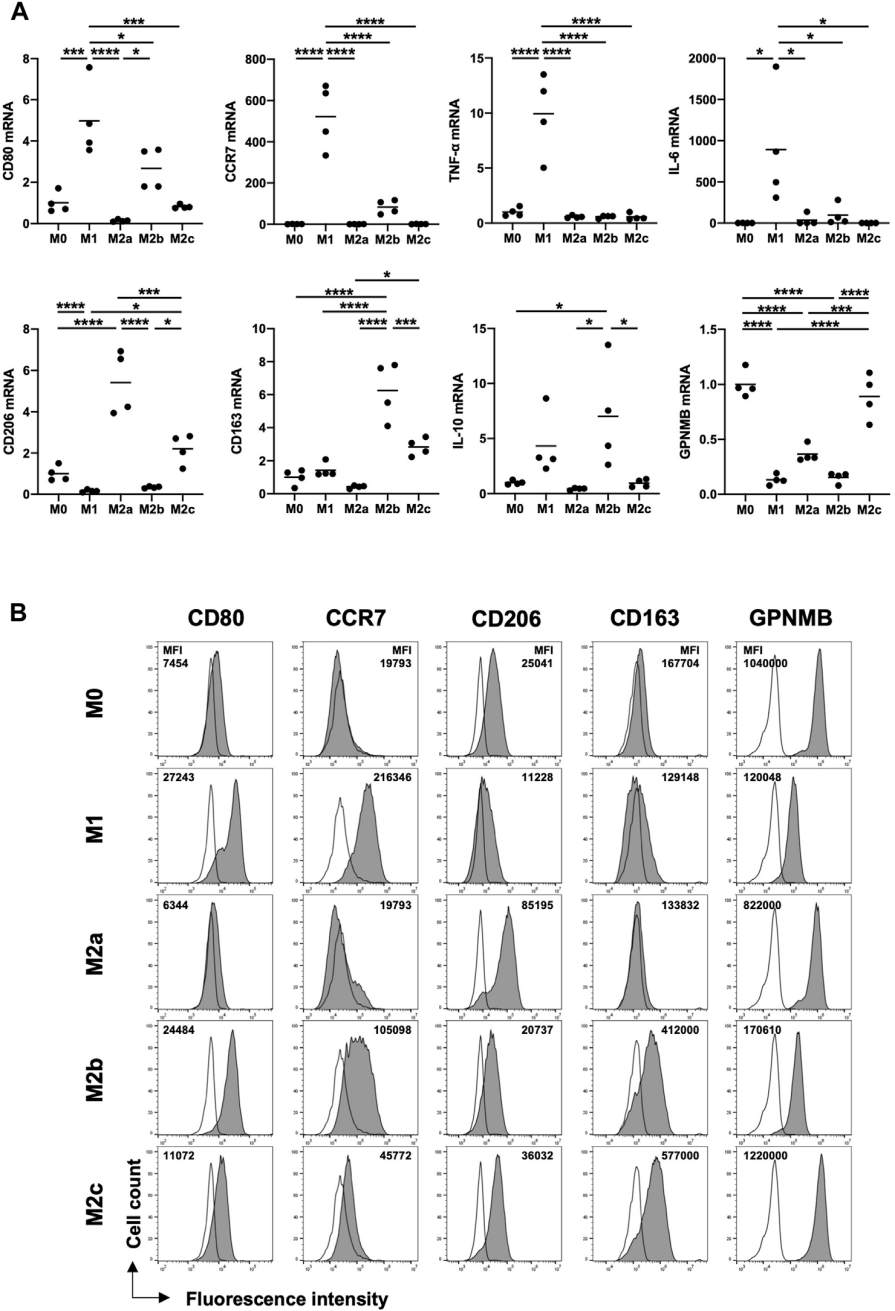
Human monocyte-derived M2c macrophages induced with IL-10 highly express GPNMB and CD163 and moderately express CD206. **(A)** mRNA expression of M1 and M2 markers in macrophages of various phenotypes from PBMCs. **(B)** Protein expression of GPNMB in macrophages of various phenotypes. RT-qPCR Ct values were normalized to *β-actin* level and expressed relative to the levels in M0, arbitrarily defined as 1. Results are presented as a mean and scatter plot of individual data points (n = 4). For flow cytometry, the cells were gated on CD68-positive cells. Filled histograms represent protein expression and open histograms represent isotype control. One-way analysis of variance, *p < 0.05, **p < 0.01, ***p < 0.001, ****p < 0.0001. GPNMB, glycoprotein non-metastatic melanoma protein B; ALI, acute liver injury; ALF, acute liver failure; HE, hepatic encephalopathy; PBMCs; peripheral blood mononuclear cells; RT-qPCR, reverse transcription quantitative PCR; IL, interleukin.

To determine the subtype of M2 macrophages expressing GPNMB and infiltrating injured liver tissues in patients with ALF and HE, we analyzed human monocyte-derived macrophages of healthy volunteers. CD80, CCR7, tumor necrosis factor-alpha (TNF-α), and IL-6 were expressed as M1 markers, especially by M1 macrophages. Regarding M2 markers, CD206 was expressed by M2a and M2c macrophages, whereas CD163 was expressed by M2b and M2c macrophages. GPNMB was expressed in M0 and M2c macrophages (Figure 4A). Next, we confirmed protein expression using flow cytometry. GPNMB was strongly expressed by M2c macrophages, which moderately expressed CD206 and strongly expressed CD163 (Figure 4B).

### 3.6 Significantly decreased CCR7 and IL-6 and increased GPNMB and CD163 expression in the PBMCs of patients with ALF

**FIGURE 5:**
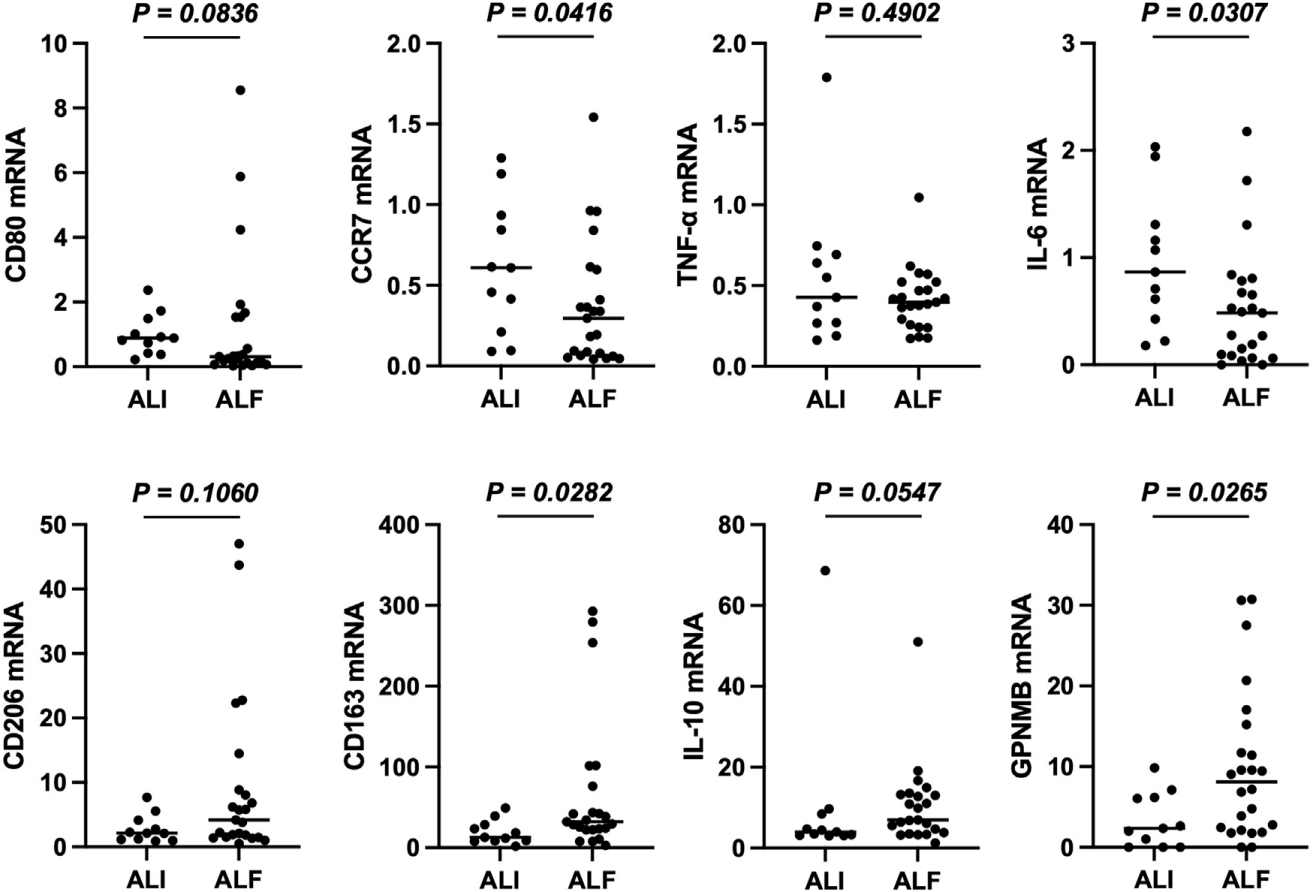
PBMCs from patients with ALF are mainly of the anti-inflammatory phenotype. mRNA expression of M1 and M2 markers in PBMCs. RT-qPCR Ct values were normalized to *β-actin* level and expressed relative to levels in a healthy volunteer, arbitrarily defined as 1. The results are presented as a median and scatter plot of individual data points. Mann–Whitney *U* test was used for statistical comparison. ALF, acute liver failure; PBMCs; peripheral blood mononuclear cells; RT-qPCR, reverse transcription quantitative PCR.

Finally, we confirmed whether the PBMCs of patients with ALF expressed GPNMB and the phenotypes of these cells. PBMCs were collected from 34 of the 56 patients and analyzed. Of these, 11 patients had ALI, 17 had ALF without HE, and six had ALF and HE. Compared with those from patients with ALI, the PBMCs of patients with ALF showed significantly increased *GPNMB* and *CD163* mRNA expression (*p* = 0.0282 and *p* = 0.0265, respectively), decreased *CCR7* and *IL-6* expression (*p* = 0.0416 and *p* = 0.0307, respectively), and a tendency for increased expression of other M2 markers and decreased expression of other M1 markers (Figure 5).

## 4 Discussion

In the present study, we found that the serum GPNMB level increased in patients with ALF, especially in those with HE. The peak serum GPNMB level correlated with disease severity and prognosis in patients with ALI and ALF, and the expression of GPNMB in M2c macrophages infiltrating injured liver tissues increased with the severity of liver injury. Moreover, we confirmed that GPNMB and CD163 were highly expressed in the PBMCs of patients with ALF compared with those in the PBMCs of patients with ALI. These findings are novel and original for the ALF category.

As GPNMB expression by infiltrating macrophages is enhanced during the recovery phase in CCl_4_-induced ALI model mice (Kumagai et al., 2015), we hypothesized that the serum GPNMB level increases after a peak in the ALT level in patients with ALI and ALF. Therefore, we defined “Day 0” as the day of peak ALT level and evaluated whether the serum GPNMB level increased in patients with ALI and ALF. The serum GPNMB level increased with ALT level or after it peaked in patients with ALI and ALF. As a reference, we previously investigated the concentration of serum GPNMB in 73 healthy control participants and found that the median serum GPNMB level was 3.1 ng/mL (data not shown). In this study, the median serum GPNMB levels in patients with ALI and ALF were 9.1 and 16.8 ng/mL, respectively. A high serum GPNMB level in patients with ALF was sustained compared with that in patients with ALI, especially when the peak GPNMB level was delayed after a peak in the ALT level in patients with ALF and HE. Prothrombin time (specifically the INR) is a universal indicator of disease severity and is strongly associated with the prognosis of patients with ALF (O’Grady et al., 1989; Robert and Chazouillères, 1996). The MELD score, which includes the INR, also predicts the outcomes of patients with fulminant hepatic failure or ALF (Kamath et al., 2001; Dhiman et al., 2007). HGF strongly induces DNA synthesis in hepatocytes (Gohda et al., 1986) and correlates with the progression of HE in patients with fulminant hepatitis, reflecting their prognosis (Tsubouchi et al., 1989). The tyrosine-protein kinase Met (cMet), which HGF binds specifically, is expressed by not only epithelial cells but also macrophages (Galimi et al., 2001). Previously, we reported that HGF-MET signaling shifts M1 macrophages toward an M2-like phenotype (Nishikoba et al., 2020). As GPNMB is expressed by M2c macrophages, we expected that there is a correlation between GPNMB and HGF levels. Actually, serum GPNMB levels were significantly correlated to not only INR and MELD score, but also HGF levels, indicating that the serum GPNMB level may have acted as a marker for the prognosis of ALI and ALF in our study.

In the present study, in the injured liver tissues of patients with ALI and ALF, GPNMB was expressed by CD68-positive macrophages, and its expression increased with the severity of liver injury, which showed M2 polarization with CD206 and CD163 expression. GPNMB expression increases in injured liver tissues of AH compared with that in normal liver tissues and is expressed by CD68-positive macrophages in cirrhotic human liver (Ramachandran et al., 2012; Harris et al., 2022), which is consistent with our findings. Furthermore, M2c macrophages strongly expressed GPNMB and CD163 and moderately expressed CD206, which is consistent with the phenotype of infiltrating GPNMB-positive macrophages in the injured liver of patients with ALF and HE. Human monocyte-derived M2c macrophages present significantly higher CD163 expression and slightly higher CD206 expression than M0 macrophages (Spiller et al., 2014; Cutolo et al., 2019). Moreover, M2c macrophages are associated with angiogenesis, especially vascular remodeling, whereas M1 macrophages promote the sprouting of blood vessels via the secretion of vascular endothelial growth factor and TNF-α, which is also required for hepatocyte proliferation (Böhm et al., 2010; Spiller et al., 2014; Lee et al., 2021). Therefore, both M1 and M2 macrophages are necessary for the regeneration of liver tissues. In our study, a few M1 macrophages were expressing CD80 or CCR7 in the injured liver tissues of patients with ALF and HE, likely due to steroid use or compensatory anti-inflammatory response syndrome (CARS) due to excessive liver injury (Antoniades et al., 2008). This result may explain the impaired liver regeneration in patients with ALF and HE.

In patients with ALF, the PBMCs were generally inclined towards M2 polarization and showed significantly increased *GPNMB* and *CD163* mRNA expression compared with that in patients with ALI. Once ALF progresses, inflammatory cytokines such as TNF-α and IL-6 are released into the bloodstream and activate inflammatory monocytes, leading to systemic inflammatory response syndrome (SIRS). Along with SIRS, inflammatory monocytes shift to pro-restorative monocytes owing to the release of anti-inflammatory mediators from the liver, and CARS develops (Triantafyllou et al., 2018). Our results reflected the CARS state in patients with ALF, and the increased GPNMB expression indicated an anti-inflammatory response. Therefore, sustained GPNMB expression translates into sustained CARS, which reflects sustained anti-inflammatory cytokines release into the bloodstream from the injured liver due to excessive liver damage. This is consistent with our findings showing that a persistently high serum GPNMB level correlated with the severity of liver injury and prognosis of patients with ALI and ALF.

Our study has several strengths. The serum GPNMB level was measured at least at three time points, whereas previous studies have not shown the dynamics of serum GPNMB levels over time. This finding is important for elucidating the implications of the GPNMB level. By assessing the changes in serum GPNMB levels over time, we found that patients with a persistently high serum GPNMB level had a poor prognosis. Additionally, only a few studies have investigated GPNMB expression and the phenotype of GPNMB-positive macrophages in injured human liver tissue (Ramachandran et al., 2012; Harris et al., 2022). Nevertheless, our study has a few limitations. This was a retrospective, single-center, observational study with a limited number of patients. We need to conduct a multicenter prospective study to clarify whether serum GPNMB level can serve as a prognostic factor. Moreover, we primarily assessed the phenotype of GPNMB-positive macrophages infiltrating injured liver tissues extracted during LT in patients with ALF and HE, as GPNMB expression in injured liver tissues was stronger in patients with ALF and HE than in patients with ALI or ALF without HE. As all patients with LT received steroids before undergoing LT, the steroids may have induced M2c macrophages (Cutolo et al., 2019; Lescoat et al., 2019). However, there were a few GPNMB-positive macrophages in patients with autoimmune hepatitis treated with steroids. As the phenotype of macrophages changes with time during liver injury (Tacke and Zimmermann, 2014), there may be a bias in the timing of liver biopsy and LT. However, we believe that human samples are precious, and it is difficult to adjust the timing of liver biopsy and LT.

In conclusion, a persistently high serum GPNMB level and its peak reflected the severity of liver injury and prognosis in patients with ALI and ALF. In addition, GPNMB was expressed in M2c macrophages infiltrating injured liver tissues. Although an increased serum GPNMB level may indicate a recovery phase during liver injury, persistently high levels may indicate impaired recovery following excessive liver injury. Thus, a persistently high GPNMB level may be a prognostic marker in patients with ALI and ALF. However, the role of GPNMB remains unclear, and further investigation is needed to determine potential clinical applications.

## Data Availability

The raw data supporting the conclusion of this article will be made available by the authors, without undue reservation.
