# Supplementary material for: Glycoprotein non-metastatic melanoma protein B expression correlates with the prognosis of acute liver injury/failure
Source: Front Cell Dev Biol. 2023 Oct 24;11:1242152. doi: 10.3389/fcell.2023.1242152 (PMC10627855; doi:10.3389/fcell.2023.1242152)
Supplement: Supplementary file 1 [file DataSheet1.docx]

Supplementary Material

# Supplementary Figures and Tables

## Supplementary Figures


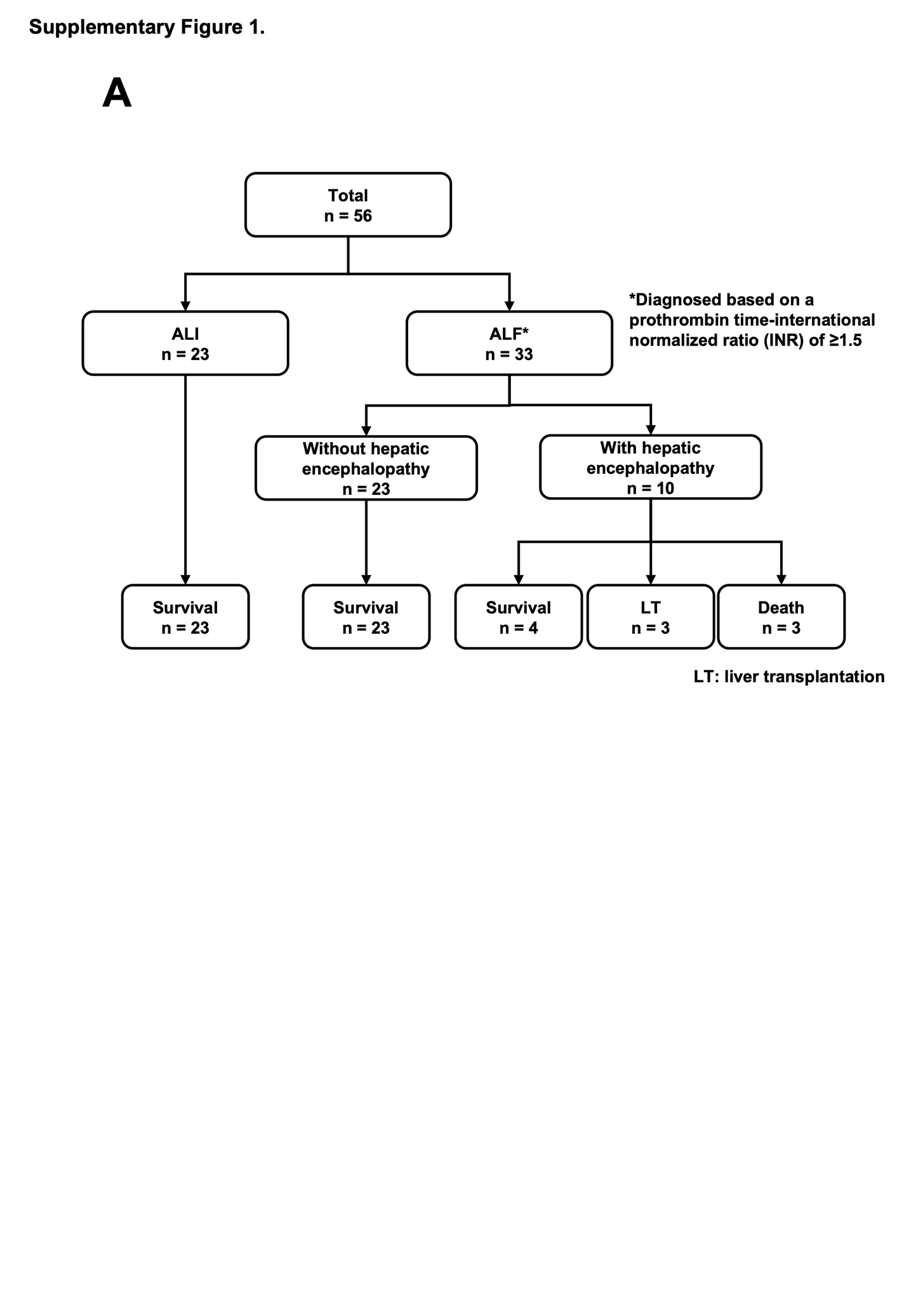


**Supplementary Figure 1.** Diagnosis and outcomes of all patients in the study. Fifty-six patients with acute liver injury (ALI) or acute liver failure (ALF) who visited Kagoshima University Hospital from June 1, 2006, to December 30, 2018, were enrolled. Their serum GPNMB levels were measured at least thrice during the observation period. ALF was diagnosed based on an INR of ≥1.5. One patient who underwent liver transplantation was included in the “Death” group owing to death after liver transplantation. GPNMB, glycoprotein non-metastatic melanoma protein B


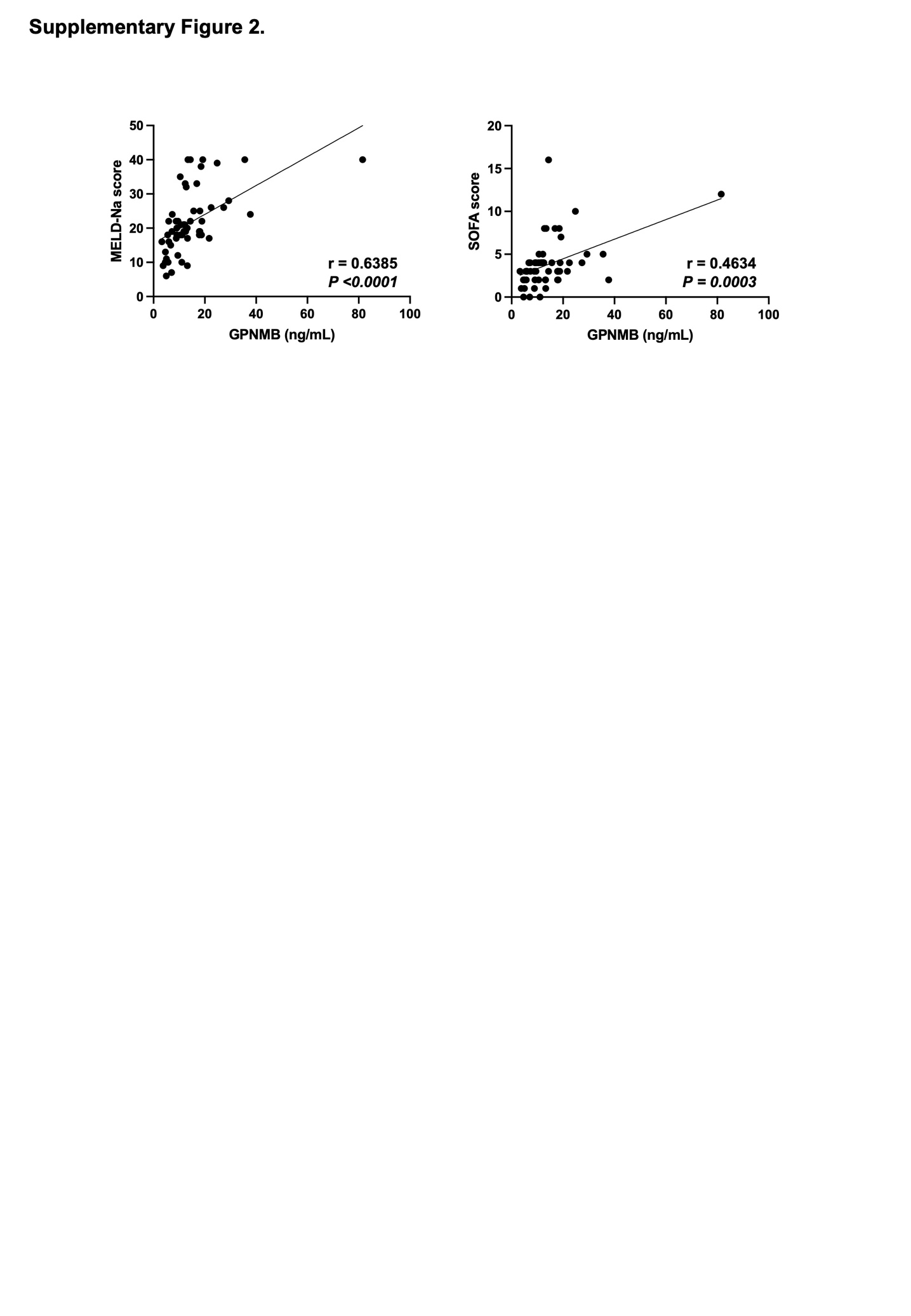


**Supplementary Figure 2.** Correlation between the peak serum GPNMB level and MELD-Na score or SOFA score. Spearman's rank-correlation coefficient was used to determine correlation. GPNMB, glycoprotein non-metastatic melanoma protein B; MELD, Model for End-Stage Liver Disease; SOFA, sequential organ failure assessment.

**
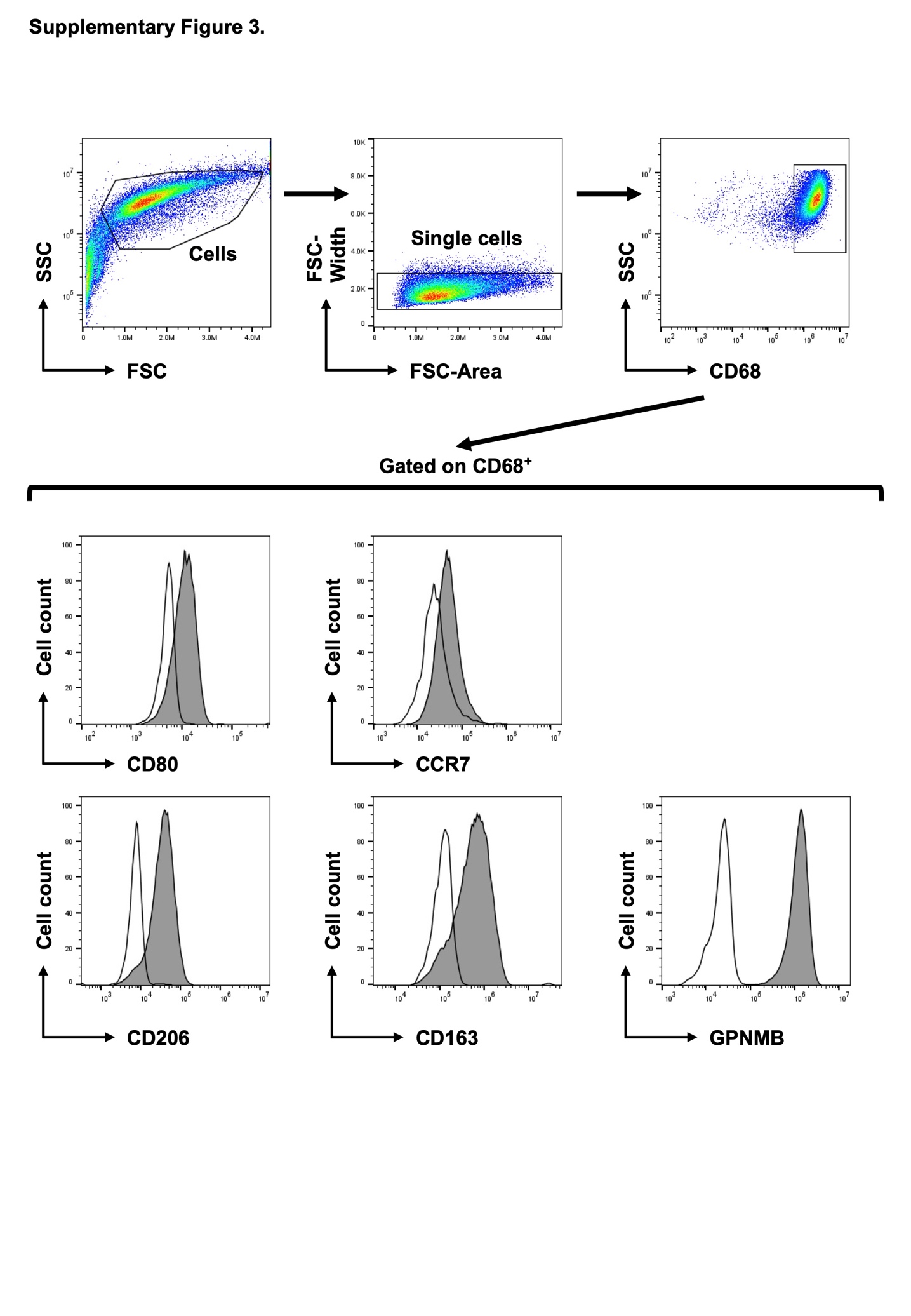
**

**Supplementary Figure 3.** Gating strategy for flow cytometry of macrophage markers. Filled histograms represent protein expression and open histograms represent the isotype control. GPNMB, glycoprotein non-metastatic melanoma protein B; SSC, side scatter; FSC, forward scatter

## Supplementary Tables

**Supplementary Table 1.** List of patients whose liver histological findings were evaluated.

**Supplementary Table 2.** Antibodies used in histology.

**Supplementary Table 3.** Primers used in RT-qPCR.

**Supplementary Table 4.** Fluorophore conjugates
